# Does the Webster’s Triangle Preservation Really Matter? A Retrospective Analysis of the Low-to-Low Osteotomies in the Head of the Inferior Turbinates

**DOI:** 10.1093/asjof/ojae079

**Published:** 2024-09-20

**Authors:** Luis C Ishida, Matheus Guedes, Guilherme F F Alves, Julia O da Fonseca, Eduardo L Fonesca, Giulia G Takahashi, Rolf Gemperli

## Abstract

**Background:**

Osteotomies are important maneuvers in the plastic surgeon’s arsenal for the nasal dorsum treatment. However, there is a concern regarding a possible medialization of the inferior turbinate’s heads and narrowing of the internal nasal valve if the Webster’s triangle is not preserved.

**Objectives:**

To analyze the mobilization of the inferior turbinates during primary rhinoplasty after lateral osteotomies.

**Methods:**

This is a retrospective study in which we analyzed 37 patients who underwent very low-to-low osteotomies in our service, during primary rhinoplasties. Axial and coronal computed tomography scans were obtained, and preoperative and 6 months postoperative images were compared with Radiant DICOM Viewer software (Medixant, Poznan, Poland) in 3-dimensional multiplanar reconstruction.

**Results:**

There were no statistically significant differences between preoperative and postoperative measurements of the lower turbinate’s osseous bases, both in axial (*P* = .305) and coronal (*P* = .08) images.

**Conclusions:**

Low-to-low osteotomies showed no medialization of the inferior turbinate’s heads or narrowing of the internal nasal valve in this study.

**Level of Evidence: 4 (Therapeutic):**

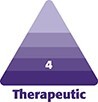

Rhinoplasty is one of the most common cosmetic procedures. It can have an aesthetic and/or functional approach, even though functional care still applies to aesthetic procedures.^1^ There is a great concern in preserving the nasal function, restructuring or preserving the internal nasal valves, the site of greatest airflow restriction.^2^

The internal nasal valve is defined by the caudal border of the lateral superior cartilages, nasal septum, nasal floor and the head of the inferior nasal turbinate.^3^ This anatomy is fundamental once lateral nasal osteotomies could potentially medialize the lower turbinate's heads and restrict airflow.^4^ This anatomy is shown in Figure 1, and these alterations can objectively be measured through computed tomography (CT) scans.^5^

**Figure 1.**
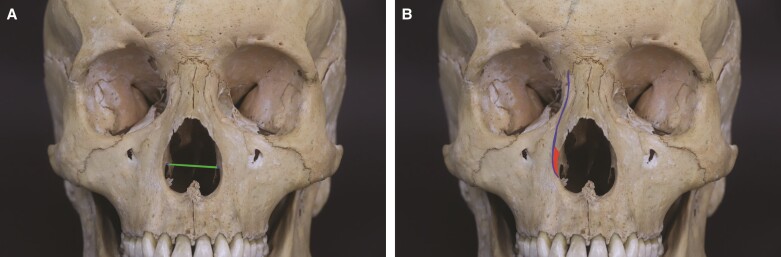
Anatomy of the pyriform opening. Cranium in frontal view. (A) Green horizontal line: distance between the lower turbinate’s osseous bases. (B) Red triangle: Webster’s triangle. Purple dot: medial canthal ligament. Blue, curvilinear line: low-to-low osteotomy pathway.

Osteotomies are an important part of the surgeon’s arsenal to correct nasal deformities and keep osseous continuity of the nasal scaffold.^6^ Its common indications include the reduction of open roof deformities, correction of lateral deviations, narrowing large dorsum, widening of narrow dorsum, and lowering the dorsum height.^7^

As it is an extremely useful procedure both aesthetically and functionally for the surgeon, Webster described in 1977 a safer way to make it. Known as a “high–low–high” osteotomy, which begins at the level of the inferior turbinate and leaves a triangular shaped bone at the piriform aperture (which would later be named Webster’s triangle) to prevent medialization of the lower turbinate's heads.^4^ Other surgeons have performed a relatively lower lateral osteotomy, known as “low-to-low,” which commences at the junction of the pyriform aperture and frontal process of the maxilla.^8^

In our practice, we perform osteotomies very down to the piriform opening, on the nasal facial recess, classifying them as “very low-to-low,” without preserving the “Webster's triangle.” In the population studied, there has been no case of internal nasal valve impairment. In search of further understanding anatomical alterations promoted by the low-to-low osteotomies, we objectively compared CT scans for alterations in the position of the lower turbinates in our patients before and after surgery.^5^

## METHODS

This is a retrospective work, in which surgical records and preoperative and postoperative CT scans of 37 patients were analyzed. The CT scans were taken before, and 6 months after surgery. All surgical procedures were made by senior residents of plastic surgery, guided by a rhinoplasty fellow and the head of the rhinoplasty group between June 2018 and February 2020. The work is also approved by our ethics committee and is in accordance with the Declaration of Helsinki.

Every patient in this study underwent a primary rhinoplasty that included very low-to-low lateral osteotomies. Secondary procedures, trauma, and severe congenital malformations were excluded from this group.

**Figure 2.**
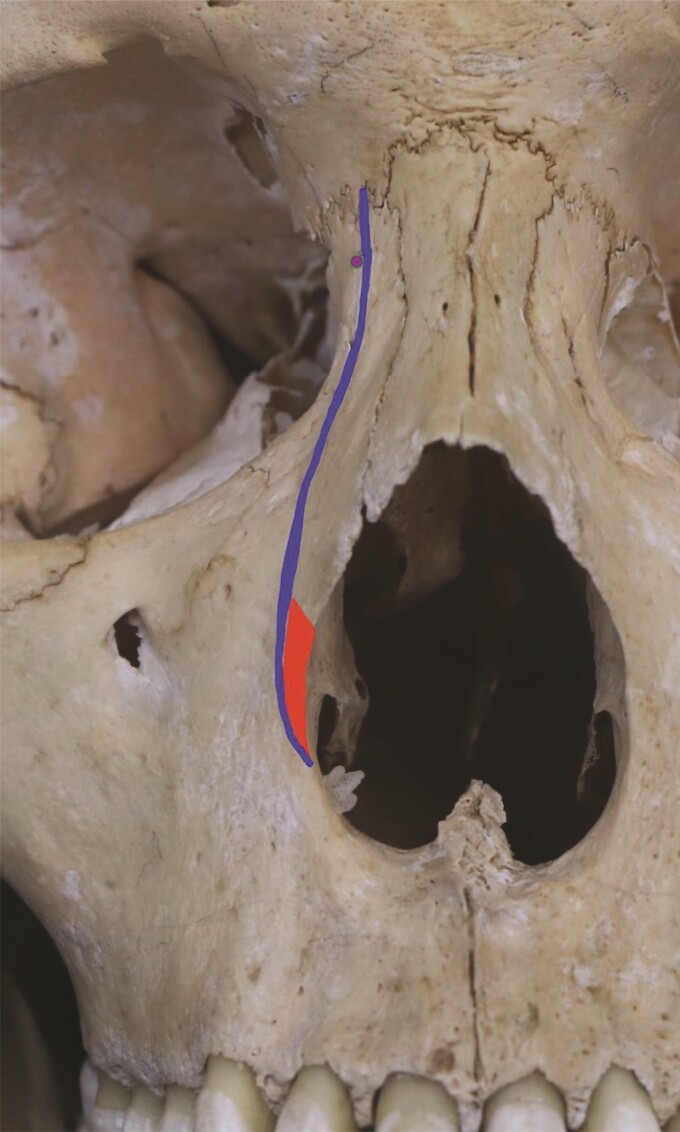
Anatomy and osteotomies sites. Cranium in frontal and oblique view. Red triangle: Webster’s triangle. Purple dot: medial canthal ligament. Blue line: low-to-low osteotomy pathway.

The surgical technique applied included lateral osteotomies lower than the commonly used “low-to-low” osteotomies. These were made with guarded straight osteotomes after subperiosteal undermining and bone sawing. Very low-to-low technique starts the fracture below the inferior turbinate’s head, in the lower portion of the lateral opening of the piriform fossa, and directs by the frontal process of the maxilla to the medial cantus direction, as illustrated in Figure 2.

The lateral nasal bones were medialized through an “in-fracture” maneuver with a greenstick fracture in the cephalic portion of these bone segments. When demanded, medial osteotomies were also performed. At the end of surgery, nasal plugs, draping, and plaster were applied.

No procedures regarding direct mobilization or resection of the lower turbinates were performed. No procedures regarding reconstruction of the internal nasal valve—such as spreader flaps or grafts—were performed in this cohort. All humps were treated with cartilaginous push down where the upper lateral and the septal portion of the internal valve are preserved.^9^

**Figure 3.**
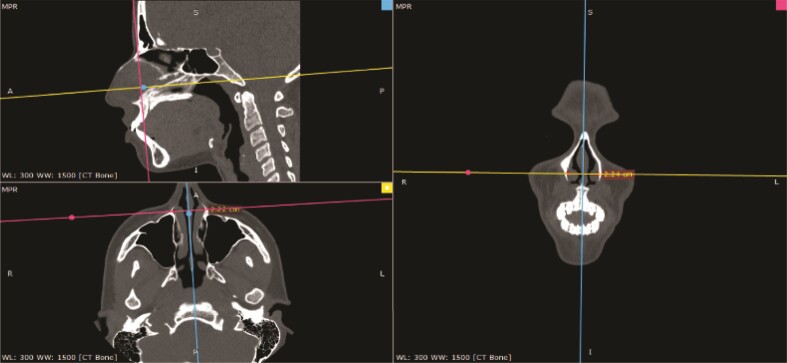
Preoperative evaluation. Preoperative computed tomography scan measure of the distance between inferior turbinate’s heads in Radiant’s software multiplanar reconstruction mode.

**Figure 4.**
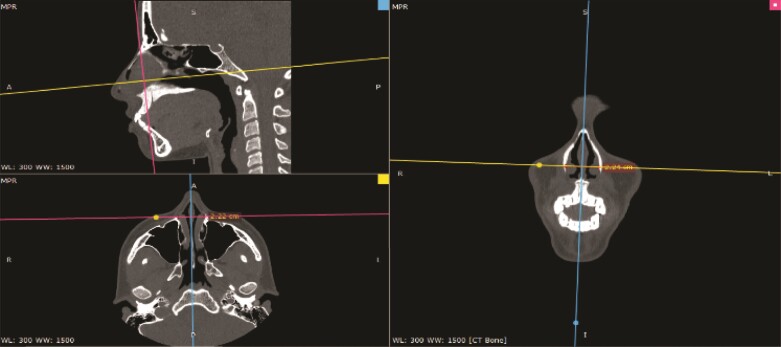
Postoperative evaluation. Postoperative computed tomography scan measure of the distance between inferior turbinate’s heads in Radiant’s software multiplanar reconstruction mode.

CT scans were analyzed through the Radiant DICOM Viewer software (Medixant, Poznan, Poland) in 3-dimensional multiplanar reconstruction. Axial and coronal images were acquired preoperatively and postoperatively, and the distance between the most anterior and lower osseous bases of the inferior turbinate’s heads were measured in centimeters (Figures 3, 4). As measures were compared, statistical analysis was performed to evaluate eventual medialization of the turbinate’s heads.

## RESULTS

Of the 37 patients, 10 were males (27.02%) and 27 were females (72.98%), with age varying from 16 to 43 years old. All were submitted to very low-to-low lateral osteotomies, and 7 (18.9%) also included medial osteotomies alongside the lateral osteotomies.

**Figure 5.**
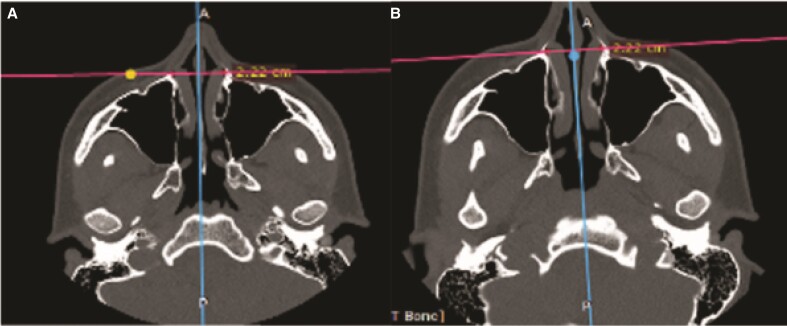
Inferior turbinates distance in axial images.

**Figure 6.**
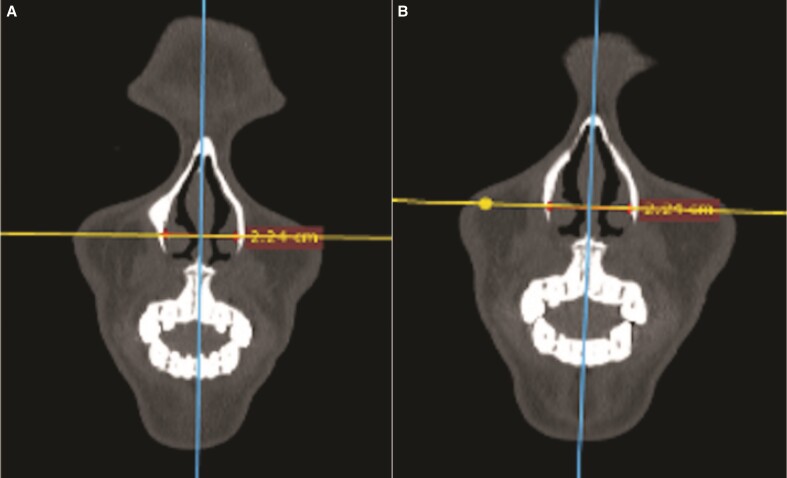
Inferior turbinates distance in coronal images.

Preoperative distance between the osseous base of the anterior portion of the inferior turbinate’s head varied from 1.75 to 2.65 cm (median of 2.09 at axial and 2.10 at coronal cuts). Postoperatively, these distances varied from 1.76 to 2.64 cm (median of 2.09 in both cuts). Measurements are displayed in Table 1 and Figures 5 and 6.

**Table 1.** Comparison of the Inferior Turbinate’s Distances

Characteristics	Measures (cm)
Preoperative	
Axial	2.09 ± 0.21
Coronal	2.10 ± 0.21
Postoperative	
Axial	2.09 ± 0.20
Coronal	2.09 ± 0.20

All data are presented as median ± standard deviation.

For descriptive analysis, quantitative measures were represented as medians and standard deviations when compatible with normality and by median and interquartile ranged when not normal, with relevant outliers. Normality definition was acquired through graphic analysis and Shapiro–Wilk’s test.^10^ Categoric variables were represented as frequency and percentages.

Concordance analysis between measures was made with a Bland–Altman’s test, in association with graphic analysis and linear regression. Paired analysis was also performed with preoperative and postoperative measurements. Analysis was performed with IBM Statistical Package for the Social Sciences software (SPSS, Chicago, IL) 20.0.

Variables were also compared by the Wilcoxon’s sign test, with no statistically significant differences between preoperative and postoperative measurements of the lower turbinate’s heads, both in axial (*P* = .305) and coronal (*P* = .08) images. The results are presented according to the second interquartile (IIQ) in Table 2.

**Table 2.** Comparative Analysis of the Inferior Turbinate’s Distances

	Pre (median, IIQ)	Post (median, IIQ)
Axial (mm)	2.1 (1.98-2.22)	2.07 (1.97-2.18)
Coronal (mm)	2.1 (1.99-2.22)	2.09 (1.97-2.19)

## DISCUSSION

Osteotomies are surgical maneuvers necessary to correct diverse nasal dorsum deformities. Historically, high–low–high fractures were chosen for their ability to preserve the Webster's triangle, avoiding medialization of the nasal turbinate's heads and subsequent narrowing of the internal nasal valves.^2^ Literature review pointed that low-to-low osteotomies would further increase this risk.^2,10,11^

However, the greater ability to medialize the lateral nasal walls with the low-to-low technique translates in a higher capacity for the surgeon to shape the nasal dorsum.^12^ Mirza et al demonstrated the safety of the technique in not worsening the patient’s respiration, as well as showing higher aesthetic satisfaction (odds ratio = 3.8, 95% CI 1.1-12.6, *P* = .03) when comparing low-to-low with low-to-high osteotomies.^13^

Literature review also reveals other ways to do osteotomies. In this sense, some authors seek to standardize the technique applied to each nasal dorsum deformity identified.^14^ Other authors showed that the nasomaxillary crease is higher than expected in relation to the maxillary depression.^15^

In our findings, the low-to-low osteotomies can be used in all cases without the risk of medializing the lower turbinate’s heads. The osseous base of the lower turbinates seems to be out of the lines of the osteotomies. Of course, an intraoperatory inspection of the lower turbinate’s heads is advisable after the osteotomies are completed. If an eventual medialization is noted, the surgeon may either mobilize or resect the lower turbinate head.

There were no statistically significant differences when comparing distances between the heads of the lower turbinates in preoperative and postoperative CT scans. Our patients also showed no clinical functional changes in respiration to this day. The nasal hump treatment we use preserves the integrity of the upper lateral and septum junction, not impairing the internal valve at this point.^9^ However, in our analysis, we did not include rhinomanometric airflow data. The airway patency was evaluated only by direct and active clinical assessment.

### Limitations

As this was a retrospective study, there was no specific evaluation regarding the respiratory functions of these patients, even though there were no clinical signs of nasal valve insufficiency.

## CONCLUSIONS

Low-to-low osteotomies showed no medialization of the inferior turbinate’s heads or narrowing of the internal nasal valve in this study.
